# Effect of steaming on chemical composition of Mediterranean mussel (*Mytilus galloprovincialis*): Evaluation of potential risk associated with human consumption

**DOI:** 10.1002/fsn3.2903

**Published:** 2022-05-05

**Authors:** Katya Peycheva, Veselina Panayotova, Rositsa Stancheva, Lubomir Makedonski, Albena Merdzhanova, Gaetano Cammilleri, Vincenzo Ferrantelli, Vittorio Calabrese, Nicola Cicero, Francesco Fazio

**Affiliations:** ^1^ Department of Chemistry Medical University Varna Varna Bulgaria; ^2^ Istituto Zooprofilattico Sperimentale Della Sicilia Palermo Italy; ^3^ Dipartimento di Scienze Biomediche e Biotecnologiche Università Degli Studi di Catania Catania Italy; ^4^ Dipartimento SASTAS Università Degli Studi di Messina Messina Italy; ^5^ Dipartimento di Scienze Veterinarie Università Degli Studi di Messina Messina Italy

**Keywords:** Black Sea, fatty acids, heavy metals, human health risk, mussel, steaming

## Abstract

Steaming process is the most popular method for cooking mussels worldwide. The effect of this cooking process on some toxic (Cd, Ni, Pb), essential (Cr, Cu, Fe, Mn, Zn) elements, minerals (Na, K, Ca, Mg), total lipids, and fatty acid profiles in the Mediterranean mussels (*Mytilus galloprovincialis*) harvested from the Black Sea was studied. Different approaches to assess the benefits and risks (*n*‐6/*n*‐3, PUFA/SFA, AI, TI, h/H, EDI, THQ, HI, TR, and HQ_EFA_) were employed. In general, steaming process significantly modified some essential elements and minerals concentrations as well as the fatty acid profiles. Compared to the raw samples, this culinary practice resulted in an increased concentration of Na, Mg, Zn, and saturated fatty acids and a decrease of polyunsaturated fatty acids. Significant changes in the lipid quality indices (PUFA/SFA, AI, TI, and h/H) from the raw samples were observed. No effect on the DHA content was found. However, the significant increase in the absolute content of EPA + DHA indicates that steaming does not compromise the nutritional quality of mussels. Target hazard quotients (THQs) and hazard index (HI) from elemental intake were below 1, indicating that the steamed *M. galloprovincialis* pose no hazard for the consumers. The target risk (TR) values for Pb, Cr, and Ni were calculated, evaluated, and showed acceptable or negligible levels. In addition, the benefit–risk ratio indicated that the steamed *M*. *galloprovincialis* are safe for human consumption.

## Graphical abstract

This study aimed at evaluating the effect of steaming process in the nutritional properties of Mytilus galloprovincialis. Compared to the raw samples, this culinary practice resulted in an increased concentration of Na, Mg, Zn, and saturated fatty acids and a decrease of polyunsaturated fatty acids. Our findings suggest that steaming could be an appropriate procedure for mussels’ consumption according to retained nutritional and healthy criteria and against potentially pathogenic microorganisms.

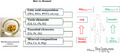

## 1 INTRODUCTION

The mussels from family Mytilidae especially *Mytilus galloprovincialis* are important and prospective species traditionally consumed in Bulgaria and Europe (Cammilleri et al., 2020). *Mytilus galloprovincialis* (Lamark, 1819) is a valuable bioresource, and the favorable conditions of Black Sea (temperature, salinity, and food availability) stimulated the mussel farming in this region. In recent years, the growing market interest in this species is based on the proven high nutritional quality and *M. galloprovincialis* have a future as a promising source of high‐quality protein, polyunsaturated fatty acids, and essential macro‐ and microelements. A few studies characterized this species as a beneficial food which could provide the well‐balanced chemical composition and through their consumption could prevent various nonchronic diseases (Merdzhanova et al., 2019; Őzden et al., 2010; Peycheva et al., 2021a). On the other hand, as filter feeding organisms they can accumulate different pathogens and contaminants. The raw mussels can deteriorate quickly and they must undergo heat treatment. Moreover, when cooking this species, it is important to regulate the temperature and time of cooking to better protect its beneficial quality. One of the suitable heat treatment methods for delicate tissues is steaming. This mild and fat‐free method can keep the food moisture and biologically active components. The cooking procedures can affect the chemical composition and cause a decrease in nutritional quality of mussel tissues. Some of the major changes which reduce the lipid quality are related to oxidation of long‐chain polyunsaturated fatty acid (PUFA) compared to saturated fatty acid (SFA) (Biandolino et al., 2021). According to Barbosa et al. (2018), the steaming process can significantly increase the content of microelements such as Cu, Pb, and Cd, and this effect is species related. Consequently, the study of the nutritional quality changes of steamed mussel tissues is more appropriate than the raw ones. Currently the benefit–risk evaluation assumed from the toxic and essential elements contents and omega‐3 PUFA levels are mostly based on the analysis of raw mussel tissues (Barbosa et al., 2018; Ghribi et al., 2021). The studies concerning this topic are mainly focused on various fish species and only few data (Barbosa et al., 2018; Biandolino et al., 2021) are available on mollusks, especially *M. galloprovincialis* as well as the benefit–risk ratio. However, to the best of our knowledge, no studies concerning the influence of steaming on toxic, essential, and mineral elements and fatty acid content of Black Sea seafood (including mollusk) have been carried out.

Hence, in this article, we had tried to study (1) the toxic, essential, and mineral elements (Cd, Cr, Cu, Fe, Mn, Ni, Pb, Zn, Ca, Mg, Na, and K) concentration and fatty acid composition of raw and steamed *M*. *galloprovincialis* farmed in Bulgarian part of the Black Sea; (2) the impact of steamed and raw *M. galloprovincialis* on human health assessed by the most commonly used risk indices (*n*‐6/*n*‐3, PUFA/SFA, AI, TI, h/H, EDI, THQ, HI, and TR) based on the average concentration of trace elements and *n*‐3 LC‐PUFAs contents that could be reached via consumption of mussels; (3) the benefit–risk ratio for human health based on trace elements and *n*‐3 LC‐PUFAs contents in mussels (HQEFA).

## 2 MATERIALS AND METHODS

### 2.1 Collection of the samples and preparation

The samples were obtained from the local mariculture farm within the northern part of Bulgarian shore of Black Sea during summer of 2021. Approximately 2–4 kg of bivalves with comparable shell length were collected, placed into bags, and brought to the laboratory in ice boxes. Two hundred specimens of each species were taken randomly for the determination of sample mean. The average length of the mussels was 4.49 ± 0.62 cm. The samples were washed with cold distilled water and were randomly divided into two groups: (1) raw and (2) steamed. Each group comprised 60 mussels with 20 individuals for each replicate (*n* = 3). The raw samples were washed with Milli‐Q water, brushed, shucked and soft tissues were removed with a Teflon knife, and stored in polyethylene bags at −20°C until analysis. The rest of the mussels were placed in a steam cooker and were steam‐cooked at 90°C for 10 min in core. After the steaming, cooked samples were placed on a filter paper to absorb the excess moisture and then the flesh was removed using a Teflon knife. Moisture content was determined on raw and steamed mussels by drying the sample in an oven at 105°C for 3 hr (AOAC, 2000).

### 2.2 Chemical analysis

Each tissue of the raw and steamed sample (around 1 g wet weight) was weighed, placed in Teflon digestion vessels. Acid wet digestions using 8 cm^3^ HNO_3_ (65% w/v) and 2 cm^3^ H_2_O_2_ (30%w/v) were performed using a microwave closed vessel digestion system MARS 6 (CEM Corporation, USA) subject to three‐stage program, maximum temperature of 210°C, ramp 15 min, pressure 800 psi, and maximum power 1050 W. The digested mollusk samples were cooled to 30°C, diluted to 25 cm^3^ with Milli‐Q water, and stored in polyethylene bottles until analysis.

The concentrations of Cd, Cr, Cu, Fe, Ni, Pb, Mn, Zn, K, Ca, Mg, and Na in the samples were determined using ICP‐OES Spectrometer (Optima 8000; Perkin Elmer) with plasma gas flow—10 L/min, auxiliary gas flow—0.7 L/min, nebulizer gas flow—0.2 L/min, peristatic pump flow rate—1.5 ml/min, processing peak—area/high, spray chamber—cyclonic glass, nebulizer—concentric glass, MEINHARD^®^, injector—alumina 2.0 mm i.d., background correction—1 or 2 point, manually, and axial or radial plasma view. The accuracy of the procedure for the determination of trace metals in mollusk was tested using ERM‐CE 278k (Mussel Tissues from European Commission, Joint Research Center, Belgium) certified reference material. The CRM was digested and analyzed in the same way as the analytical samples. The recovery values were between 86% and 103% for the individual elements.

### 2.3 Estimated daily intake, target hazard quotient, and target risk calculations for toxic metals

For the calculation of EDI, the following formula was used:

$$EDI=\frac{M_{c}x\;IR}{BW_{a}}$$

where *EDI* is the estimated daily intake (mg toxic/essential element/kg body weight/day), *FIR*—average daily consumption of mollusks (kg/person), *M_c_*—average concentration of toxic/essential metal (mg/kg), and *B_w_*—body weight (kg). Mussel consumption rate in Bulgaria was 0.8 g/capita/day in 2013 (FAO, 2020) and 60 kg is the average body weight used for adults (Zhelyazkov et al., 2018).

US EPA Region III Risk‐Based Concentration Table (USEPA, 2020) was used to calculate the *THQ* by the ratio of exposed toxic/essential element concentration to the reference dose concentration and gives information about the long‐term noncarcinogenic exposure probabilities.

$$THQ=\frac{\left(M_{C}\;.\;I_{R\;}.\;10^{‐3}.\;EF\;.\;ED\right)}{\left(RfD\;.BWa\;.\;ATn\right)}$$

where *M_C_* is the metal concentration in mussel species (mg/kg w.w.), *I_R_* is the average daily consumption of mussels (g/day) (0.8 g/person/day) (FAO, 2020), *EF* is the exposure frequency (365 days/year), *ED* is the exposure duration (70 years) for noncancer risk as used by USEPA (2020), *RfD* is the reference oral dose of individual element (1 × 10^−3^ mg kg^‐1^ day^‐1^ for Cd, 3 × 10^−3^ mg kg^‐1^ day^‐1^ for Cr, 4 × 10^−2^ mg kg^‐1^ day^‐1^ for Cu, 7 × 10^−1^ mg kg^‐1^ day^‐1^ for Fe, 2 × 10^−2^ mg kg^‐1^ day^‐1^ for Ni, 2 × 10^−3^ mg kg^‐1^ day^‐1^ for Pb, and 3 × 10^−1^ mg kg^‐1^ day^‐1^ for Zn) (USEPA, 2020), *B_w_* is the adult body weight (average 60 kg), and *AT_n_* is the average time for noncarcinogens (ED × 365 days/year) (USEPA, 2020). Values of THQ below 1 show no harmful effect for human health.

Hazard index (*HI*) is used to estimate the combined effect of contaminants and it was calculated using the formula (USEPA, 2020):

$$HI=\sum_{i}{\mathrm{THQ}}_{i}$$

Target cancer risk (*TR*) indicates carcinogenic risks an individual to develop cancer over a lifetime exposure to potential carcinogens (USEPA, 1989) and it is calculated as:

$$TR=\frac{\left(M_{C}\;.\;I_{R\;}.\;10^{‐3}.\;CPSo.EF\;.\;ED\right)}{\left(BWa\;.\;ATc\right)}$$

where *CPS_o_* is the carcinogenic potency slope oral (risk per mg/kg/day) with values for Ni =1.7 mg/kg bw‐day, Cr =0.5 mg/kg bw‐day, and Pb =0.009 mg/kg bw‐day; *AT_c_* is the averaging time carcinogens (25,550 days) (EFSA, 2009, 2010, 2015a; USEPA, 2020). TR value for intake of Cr, Ni, and Pb was calculated to indicate the carcinogenic risk since Cd, Cu, Fe, Mn, and Zn do not cause any carcinogenic effects. The acceptable values of TR are 1 × 10^−6^ according to USEPA (2020).

### 2.4 Fatty acid analysis and nutritional quality indices

Total lipids (TL) were extracted from *M. galloprovincialis* using Bligh and Dyer procedure (Bligh & Dyer, 1959). Three replicates of 20 mussels each (raw and steamed) were homogenized with a laboratory blender (Isolab Laborgeräte GmbH Co., Eschau, Germany). Tissue homogenates (3 g) were extracted with chloroform/methanol (1:2 v/v), chloroform/methanol (1:1 v/v), and chloroform, followed by constant mixing for 30 min after each extraction step. NaCl solution in H_2_O (0.9% w/v) was added to the pooled extracts for phase separation. After centrifugation (3500*g*, 15 min), the bottom organic layer was collected with a Pasteur pipette, filtered through Na_2_SO_4_, and the solvent evaporated to dryness by rotary evaporator at 40°C. The dry residues were weighed and the amounts of total lipids were determined gravimetrically.

The fatty acids (FAs) of *M. galloprovincialis* were determined as fatty acid methyl esters (FAMEs) after direct transmethylation with 2% sulfuric acid in methanol (Christie, 1993). FAMEs were analyzed by gas chromatography using a Thermo Fisher Scientific FOCUS chromatograph equipped with a TRACE TR‐5MS capillary column (30 m × 0.25 mm × 0.25 µm) and a PolarisQ ion trap mass spectrometer. The oven temperature was programmed from an initial oven temperature of 40°C for 4 min, followed by a rate of 20°C/min from 40°C to 150°C and raised from 150°C to 235°C at a rate of 5°C/min, and then from 235°C to 280°C a rate of 10°C/min for 5 min. The carrier gas used was helium with a flow rate of 1 ml/min. Fatty acid identification was performed by comparing their respective retention time and mass spectrum with MS spectra of the commercial 37 Component FAME Mix standards C4–C24 and PUFA n3 from Menhaden oil (Supelco Analytical) under the same conditions of FAMEs. Individual FAs were expressed both as a percentage (%) of the total amount of fatty acid. The results for EPA and DHA were calculated as mg/100g w.w. using the corresponding conversion factors (XFA) for mollusks, proposed by Weihrauch et al. (1977).

Three nutritional quality indices were applied to assess the nutritional potential of raw and steamed mussels:

- atherogenicity index (AI) (Ulbricht and Southgate, 1991):

$$AI=\frac{C12:0+(4\times C14:0)+C16:0}{\sum {\mathrm{PUFA}}_{n‐6}+\sum {\mathrm{PUFA}}_{n‐3}+\sum MUFA}$$

- thrombogenicity index (TI) (Ulbricht and Southgate, 1991):

$$TI=\frac{C12:0+C14:0+C16:0}{\left(0.5\times {\mathrm{PUFA}}_{n‐6}\right)+\left(3\times {\mathrm{PUFA}}_{n‐3}\right)+\left(0.5\times MUFA\right)+\left(\frac{{\mathrm{PUFA}}_{n‐3}}{{\mathrm{PUFA}}_{n‐6}}\right)}$$

- hypocholesterolemic to hypercholesterolemic ratio (h/H) (Santos‐Silva and Bessa, 2002)):

$$h/H=\frac{C18:1n‐9+C18:2n‐6+C18:3n‐3+C20:4n‐6+C20:5n‐3+C22:6n‐3}{C14:0+C16:0}$$

### 2.5 Hazard quotient for benefit–risk ratio

Gladyshev et al. (2009) proposed a formula for the benefit–risk ratio of the consumption of marine organisms based on the content of LC‐PUFA and toxic/essential elements. It is estimated through calculation using the following equation:

$${\mathrm{HQ}}_{EFA=}\frac{R_{\mathrm{EFA}}.C_{\mathrm{element}}}{C.RfD.B_{w}}$$

where *R_EFA_* is the recommended daily dose of essential fatty acids (EFA) for a person (mg/day), *C_element_* is the concentration of the essential/toxic element (mg/kg), C is the content of EFA (EPA + DHA) in a given bivalve (mg/g), *RfD* is the reference dose (mg/kg/d), and *B_w_* is the average adult body weight (70 kg). A value of HQ_EFA_ less than 1 means the health benefit from bivalve consumption and HQ_EFA_ more than 1 means the risk (Gladyshev et al., 2009). For the calculation of this equation, R_EFA_=500 mg/day (EFSA, 2012, 2015b) has been used and the values of RfD were obtained from the EPA Region III Risk‐Based Concentrations summary table (USEPA, 2020), with the exception of Pb (Hang et al., 2009).

### 2.6 Statistical analysis

All analyses were performed in triplicate and the results were expressed as mean ±standard deviation (*SD*). The results for toxic, essential, and mineral elements were stated as mg/kg w.w. and for individual fatty acids as the percentage of the total fatty acids in the total lipids. *T* test was used to compare the results for heavy metals, minerals, and fatty acid composition. Differences at *p* ≤.05 were considered significant (Graph Pad Prism 6).

## 3 RESULTS AND DISCUSSION

### 3.1 The levels of toxic and essential elements and mineral content in raw and steamed samples

The mean concentrations (mg/kg w.w.) of toxic elements in the studied edible portion of *M*. *galloprovincialis* are presented in Table 1.

**TABLE 1:** Trace elements composition (mean ±standard deviation in mg/kg) of raw and steamed *Mytilus galloprovincialis* from Black Sea (Bulgaria)

	Raw	Steamed	Regulations
Toxic elements			
Cd	0.355 ± 0.05	0.384 ± 0.08	1.0 mg/kg^a^
Ni	0.033 ± 0.01	0.052 ± 0.02	–
Pb	0.046 ± 0.02	0.037 ± 0.01	5.0 mg/kg^b^
Mineral composition			
Na	1986.3 ± 212.1	2655.8 ± 152.3*	–
K	1949.1 ± 67.4	1991.3 ± 99.1	–
Ca	492.9 ± 163.8	694.4 ± 15.0	–
Mg	413.2 ± 21.4	546.7 ± 61.8*	–

*Differ significantly (*p* <.05) (raw versus. steamed).

^a^ Obtained from Zhelyazkov et al. (2018).

^b^ Obtained from EU (2006).

As it can be seen from Table 1, the highest concentration of Cd was observed in the steamed sample (0.384 mg/kg w.w.). Kalogeropoulos et al. (2012) had found values of Cd between 342 ± 146 ng/g f.w. for raw and up to 1300 ± 1141 ng/g f.w. for pan‐fried mussels from Saronikos Gulf, Attica, Greece. Amiard et al. (2008) showed that Cd content was lowered after cooking of some mollusks from France, the United Kingdom, and Hong Kong. Tengku Nur Alia et al. (2020) reported values of 0.0007 ± 0.0021 mg/kg w.w. of raw and 0.0003 ± 0.0003 mg/kg w.w. of farmed sea bass (*Lates calcarifer*) collected from Southeast Asian region. Steam used during the thermal process leads to protein degradation and loss of weight and water, so the chemical contaminants might be also affected by the applied steam (Domingo, 2011).

Kalogeropoulos et al. (2012) reported values for Ni between 0.34 ± 0.29 μg/g f.w. (raw) and 0.82 ± 0.22 μg/g f.w. (pan‐fried) mussels from Greece. Nickel concentration in *M. galloprovincialis* sampled from Boka Kotorska Bay, Montenegro ranged between 0.27 and 1.14 mg/kg w.w. (Peroševic et al., 2018) and between 1.35 and 7.05 mg/kg d.w. in the soft tissues of wild and farmed *M. galloprovincialis* collected from the Boka Kotorska Bay (Jović & Stanković, 2014).

Lead levels in mussel samples from Saronikos Gulf, Attica, Greece varied between 0.68 ± 0.11 μg/g f.w. (raw) and 1.3 ± 0.3μg/g f.w. (cooked). Tepe and Süepe (2016) in their study on *M*. *galloprovincialis* from Giresun coast, Black Sea, Turkey found values between 2.69 and 3.85 μg/g w.w., while Tengku Nur Alia et al. (2020)—0.013 ± 0.010 mg/kg for raw fish and 0.001 ± 0.003 mg/kg for cooked one.

The concentration of the toxic elements (Pb and Cd) did not exceed the maximum levels set by the European Commission Regulation No. 1881/2006 (EU, 2006). There is no maximum permitted limit for Ni set by the EU concerning bivalve species.

Table 1 also illustrates the mineral composition of the analyzed samples. The data express in it are within the range of values reported for *M. galloprovincialis* from other seas (Astorga España et al., 2007; Çelik & Oehlenschläger, 2007; Jureša & Blanuša, 2003; Keskin et al., 2007; Őzden et al., 2010; Stanković et al., 2011).

Table 2 shows the dietary intake of essential metals based on the estimated daily intake for both steamed and raw samples through consumption of 100 g of steamed/ raw mussels and the percent coverage of the recommended tolerable metal intakes.

**TABLE 2:** Essential trace element content (mean ±standard deviation) and nutritional contribution of raw and steamed *Mytilus galloprovincialis* from Black Sea (Bulgaria)

		Raw			Steamed		
	RDA or TDI (mg/day)	mg/kg	EDI	% DRI	mg/kg	EDI	% DRI
Cr	0.05 (WHO, 2008)	0.092 ± 0.02	0.001	18.40	0.117 ± 0.04	0.002	23.40
Cu	2 (EU, 2008)	0.525 ± 0.07	0.007	2.63	0.738 ± 0.09*	0.010	3.69
Fe	12.5 (NRC, 1989)	34.829 ± 6.75	0.464	27.86	35.623 ± 11.18	0.470	28.18
Mn	1.8–2.3 (IM, 2001)	0.966 ± 0.13	0.0013	5.37	1.070 ± 0.09	0.014	5.94
Zn	12 (NRC, 1989)	14.204 ± 1.67	0.189	11.84	23.540 ± 4.42*	0.314	19.62

Note

Abbreviations: % DRI, calculated percent from the daily recommended intake; AI, adequate intake; EDI, estimated daily intake; RDA, recommended daily allowances; TDI, tolerable daily intake.

*Differ significantly (*p* <.05) (raw versus. steamed).

Chromium concentration had been reported with values between 2.0 and 2.4 mg/kg d.w. by Desideri et al. (2010), between 1.022 and 12.199 mg/kg for sample of mussels caught from Bosporus of the Sea of Marmara in 2006 and 2007 (Őzden et al., 2010). The concentration level for this element in the studied species was below the range stated by several authors (Liu et al., 2019; Türkmen et al., 2005; Usero et al., 2005).

The copper level concentration varies from 0.525 ± 0.07 up to 0.738 ± 0.09 mg/kg. Őzden et al. (2010) measured the copper content in different seasons in *M*. *galloprovincialis* between 0.839 ± 0.049 mg/kg w.w. and 3.116 ± 0 0.052 mg/kg w.w., relatively high amount of Cu (719.48 μg/g d.w.) was established in a sample of mollusk from the coast of Xiangshan Bay, China (Liu et al., 2019). Kalogeropoulos et al. (2012) found that pan‐fried mussels (3.4 ± 0.3 μg/g) have a greater amount of this element compared with the raw ones (1.4 ± 0.2 μg/g).

In our previous study, Fe concentration in samples of *M*. *galloprovincialis* ranged between 119.94 ± 3.46 mg/kg w.w. and 188.24 ± 5.51 mg/kg species (Peycheva et al., 2021). Kalogeropoulos et al. (2012) had reported values for Fe from 43 ± 11 μg/g for raw samples of *M*. *galloprovincialis* up to 107 ± 32 μg/g in pan‐fried ones. The results from this study are within the data stated in the literature (Desideri et al., 2010; Jović & Stanković, 2014; Őzden et al., 2010).

Mn is an essential element for humans and the data obtained from the current study are within the range stated in the literature (Çullaj et al., 2006; Gustily & Zhang, 2002; Locatelli, 2003; Ramsak & Scancar, 2012; Scancar et al., 2007).

The FAO (1983) set a limit daily human intake for Zn 30 mg/kg. The established maximum level for zinc in Bulgarian legislation above which bivalves' consumption is not permitted is 200 mg/kg (Anonymous, 2004). The values between 3.20 ± 0.017 mg/kg for boiled and 9.68 ± 0.22 mg/kg for raw *Oncorhynchus mykiss* obtained from Antalya, Turkey has been reported (Gokoglu et al., 2004) and between 40 ± 11 μg/g for raw samples of *M*. *galloprovincialis* and 104 ± 45 μg/g for pan‐fried samples of the same species (Kalogeropoulos et al., 2012). According to the data, the analyzed samples are within the health legislation levels.

### 3.2 Dietary intake of essential elements. Effect of cooking on mussels' trace elements content

According to the data presented, the consumption of 100 g of cooked mussels by a normal 60 kg person covers a slightly significant fraction of the RDA or TDI of Cu and Mn (Table 2). Nevertheless, the cooked mussels show as a good source of Fe, with the level of 0.47 g meal reaching almost 28.18% of the required DRI, Cr (23.40%), and Zn (19.62%).

The concentration of toxic and essential elements and minerals in steamed mussels was higher than those of raw samples. The data in the literature are contradictory regarding the correlation between trace element content in raw and cooked sea shellfish. The type of cooking (baking, grilling, frying, microwave heating, steaming, sous vide) as well as the size of marine species affect the level of toxic and essential elements before and after the thermal processing. Gokoglu et al. (2004) found that Na, K, P, Mg, Zn, and Mn contents of boiled fish decreased and the authors stated that this cooking process is not appropriate for fish preparation. When the samples were grilled, the levels of Mg, Zn, and Mn decreased, while that of potassium increases (Gokoglu et al., 2004). Kalogeropoulos et al. (2012) concluded that domestic pan‐fried and grilling of shellfish from Mediterranean sea resulted in cooked products with higher metal concentration compared with the uncooked samples and the cooked species are good source of Cr, Zn, and Fe, while the toxic elements under study do not pose health risk according to the various health coefficient calculated in that work.

### 3.3 Effect of cooking on total lipid content and fatty acid composition and nutrition quality

Upon cooking, significant changes in the moisture content, the total lipid content, and the percentages of fatty acids are observed (Table 3). Total lipid content increased significantly by 25.9% on a wet weight basis. The raw and steamed mussels showed higher moisture and lower lipid content than those reported by other authors (Biandolino et al., 2019, 2021; Kalogeropoulos et al., 2012; Merdzhanova et al., 2019; Prato et al., 2019, 2020).

**TABLE 3:** Fatty acid composition of raw and steamed *Mytilus galloprovincialis*

	Raw		Steamed	
	%	*SD*	%	*SD*
Dry weight	17.21	0.08	21.13*	0.14
Total lipids (g/100 g w.w.)	1.39	0.04	1.75*	0.09
C14:0	2.16	0.18	3.00*	0.19
C16:0	18.17	0.37	23.05*	1.05
C18:0	4.36	0.53	4.30	0.27
C20:0	0.12	0.04	tr	
C22:0	tr		0.13	0.02
C24:0	0.30	0.16	0.23	0.05
SFA	**25.29**	**0.19**	30.90*	**1.36**
C16:1	6.07	0.14	7.58*	0.75
c‐C18:1n−9	1.16	0.13	1.70*	0.14
C20:1	0.56	0.17	0.33	0.10
C24:1	0.15	0.06	0.36*	0.03
MUFA	**8.12**	**0.28**	10.15*	**0.80**
C18:2n−6	0.82	0.02	1.05*	0.07
C18:3n−3	2.11	0.05	2.12	0.45
C20:2	0.38	0.12	0.41	0.03
C20:3n−6	1.56	0.92	1.61	0.27
C20:3n−3	0.18	0.10	0.35*	0.04
C20:4n−6	3.94	2.09	2.41	0.79
C22:2	0.99	0.41	nd	
C20:5n−3, EPA	7.39	0.66	9.92*	0.98
C22:6n−3, DHA	45.07	3.88	39.83	1.68
C22:5n−3, DPA	3.51	0.90	0.87*	0.26
C22:4n−3	0.63	0.32	0.32	0.03
*n*−6 PUFA	**7.70**	**1.80**	**5.48**	**0.45**
*n*−3 PUFA	**58.89**	**1.87**	**53.46**	**1.45**
PUFA	**66.59**	**0.11**	58.95*	**2.14**

Note

Results represent mean values ± standard deviation (*n* = 3).

Abbreviations: C17:1; C21:1); MUFA, monounsaturated fatty acids (including trace amounts of C14:1; nd, not detected; PUFA, polyunsaturated fatty acids; SFA, saturated fatty acids (including trace amounts of C12:0; C15:0; C17:0; C21:0; C23:0); tr, trace levels were <0.1% of TFA.

*Differ significantly (*p* <.05) (raw vs. steamed).

The raw *M. galloprovincialis* presented a favorable fatty acid composition with the predominance of PUFA (66.6%), followed by SFA (25.3%) and MUFA (8.1%). These data are in agreement with those reported previously for the same species from the Black Sea (Merdzhanova et al., 2019; Panayotova et al., 2021; Peycheva et al., 2021a, 2021b; Stancheva et al., 2017; Stratev et al., 2017). However, other authors reported that SFA was the most abundant fatty acid group in raw *M. galloprovincialis* from the Mediterranean Sea (Biandolino et al., 2019, 2021; Prato et al., 2019, 2020). As observed for the raw mussels, the PUFA fraction was the highest, followed by SFA and MUFA. Thermal treatment can cause changes in the lipid quality of products by destruction of vitamins, pigments, and fatty acids (Sampels, 2015). Steaming process seemed to cause a significant increase in SFA and MUFA contents, mainly due to the changes in C14:0, C16:0, C16:1, C18:1, and C24:1 levels. On the other hand, a significant decrease in the total PUFA content was induced during the cooking process. For *n*‐6 PUFA, the steaming resulted in a significant increase in the C18:2n‐6 levels, but had no overall effect on the total content of *n*‐6 PUFA (Table 3). The effects of the cooking process on *n*‐3 PUFA mainly involved a significant loss of DPA and thus a significant decrease in total *n*‐3 PUFA, while EPA appeared to increase. No significant differences were found for the DHA levels alone and the total amount of *n*‐3 PUFA.

Lipid quality indices of raw and steamed *M. galloprovincialis* are given in Table 4.

**TABLE 4:** Lipid quality of raw and steamed *Mytilus galloprovincialis*

	Raw	Steamed
*n*−6/*n*−3	0.13 ± 0.06	0.10 ± 0.02
PUFA/SFA	2.63 ± 0.02	1.91 ± 0.16*
DHA + EPA	543.47 ± 46.63	693.02 ± 34.10*
AI	0.36 ± 0.01	0.51 ± 0.04*
TI	0.13 ± 0.01	0.17 ± 0.02*
h/H	2.98 ± 0.15	2.20 ± 0.17*

Note

Results represent mean values ±standard deviation (*n* = 3).

*Differ significantly (*p* <.05) (raw vs. steamed).

Considering the changes in *n*‐6 and *n*‐3 PUFA, the *n*‐6/*n*‐3 ratio exhibited no significant changes during the cooking process. The *n*‐6/*n*‐3 ratio is used as an important index for the nutritional quality of marine lipids. Maintaining a low *n*‐6/*n*‐3 ratio (<1) in the everyday diet plays a crucial role in the prevention of diet‐induced diseases (Simopoulos, 2013). PUFA/SFA ratio is used to describe the quality of dietary lipids. In our study, PUFA/SFA ratio decreased significantly by 27%: from 2.63 to 1.91. According to the Department of Health (2004), the recommended minimum value of PUFA/SFA ratio is 0.45 (National Institutes of Health). Although the steaming process induced a significant reduction in PUFA content, cooked mussels seem to preserve a well‐balanced and favorable PUFA/SFA ratio (>0.45). PUFA/SFA index does not consider the dietary importance of MUFA. Therefore, the potential health beneficial effects of *M. galloprovincialis* lipids were additionally assessed by other three indices: atherogenicity index, (AI) thrombogenicity index (TI) (Ulbricht and Southgate, 1991), and hypocholesterolemic to hypercholesterolemic ratio (h/H) [30]. These indices take into account the effects that different dietary fatty acids might exert on the probability of increasing the incidence of cardiovascular diseases. The raw and steamed *M. galloprovincialis* lipids exhibited beneficial antiatherogenic, antithrombogenic, and hypocholesterolemic indices (AI, TI < 1, and h/H > 1). All lipid quality indices were significantly affected by the cooking process. The AI and TI increased by 41.7% and 30.8%, while h/H decreased by 26.2%. An inverse effect of boiling on lipid quality indices of *M. galloprovincialis* from the Ionian Sea was reported by Biandolino et al. (2021). According to Ghribi et al. (2017), AI and TI values of *Arca noae* lipids were not altered significantly after steaming or boiling.

The sum of EPA+DHA is one of the most important nutritional quality index of seafood lipids. These two fatty acids play major roles in a number of metabolic processes in the human organism (Méndez et al., 2017). According to the published data, the EPA + DHA content of mussels lipids is usually higher than that of other shellfish species (Tan et al., 2020). In this study, no significant changes were observed in the DHA expressed as a percentage of the total fatty acids. However, in order to provide useful information about the nutritive value of steamed mussels, the results for EPA+DHA are expressed as mg per 100g on a wet weight basis (edible portion). The sum of EPA and DHA significantly increased after steaming: from 543.47 mg.100 g^−1^ to 693.02 mg.100 g^−1^ w.w. (by 27.5%). A number of recommendations regarding the dietary intake of EPA+DHA have been developed by various national and international health organizations (FAO/WHO, 2003; EFSA, 2015b; Martin, 2001). The recommendations for adults vary between 250 mg/day (EFSA, 2015b) to 500 mg EPA+DHA/day (FAO/WHO, 2003). Increased intake, 1 g/day of EPA+DHA is advised for patients with coronary heart disease and an addition of 100–200 mg/day (DHA only) for pregnant or lactating women (EFSA, 2015b; FAO/WHO, 2003). In order to meet the daily requirements, a healthy individual needs to consume only 46–92 g raw or 36–72 g steamed mussels. The EPA+DHA content of *M. galloprovincialis* in this study is much higher than many other mollusk species (Tan et al., 2020).

### 3.4 Estimated target hazard quotient, hazard index and target cancer risk

Target hazard quotient (THQ), hazard index (HI), and target cancer risk (TR) are calculated using the formulas described in Section 2.3 and presented in Table 5. THQ is a coefficient which assesses the risk associated with the intake of contaminated *M. galloprovincialis*. The values below 1 (THQ < 1) reveal a lower level of exposure, which is associated with a daily exposure at this level is not likely to cause any harmful effects for human health during a lifetime in population (Bogdanović et al., 2014). According to our study, there were no THQ or HI values exceeding the value of 1 through the consumption of steamed *M. galloprovincialis* from Black Sea (Bulgaria) and it may be concluding that all tested elements in the steamed samples indicate that no health risk is present according to regulation or literature data (Jović & Stanković, 2014; Kalogeropoulos et al., 2012; Nekhoroshkov et al., 2021; Peycheva et al., 2021a, 2021b).

**TABLE 5:** Estimated target hazard quotient (THQ), hazard index (HI), and target risk (TR) for toxic and essential elements by the consumption of steamed *Mytilus galloprovincialis* from Black Sea (Bulgaria)

Element	RfD^a^ (mg/kg/d)	Estimated target hazard quotient (THQ)	CSFo (mg/kg/d)	Estimated target risk (TR)
Cd	0.001	0.0051 (0.0047)	NA	NA
Cr	0.003	0.0005 (0.0004)	0.5	7.8 × 10^−7^ (6.1 × 10^−7^)
Cu	0.040	0.00025 (0.00018)	NA	NA
Fe	0.700	0.00067 (0.00066)	NA	NA
Ni	0.020	0.00002 (0.00002)	1.7	8.4 × 10^−7^ (7.5 × 10^−7^)
Pb	0.0035	0.0002 (0.00002)	0.0085	6.2 × 10^−9^ (5.5 × 10^−9^)
Zn	0.300	0.0010 (0.0006)	NA	NA
HI	**–**	**0.008**		

Note

The values of the raw samples are given in parentheses.

^a^ Oral reference doses (RfD) were obtained from the EPA Region III Risk‐Based Table (USEPA, 2020).

TR values are calculated for intake of those heavy metals, which are considered cancerogenic (Cr, Ni, and Pb) according to IARC (2012). Based on US EPA methods, cancer risk lower than 10^−6^ is considered to be negligible, >10^−4^ is considered unacceptable, and in the range from 10^−4^ to 10^−6^ is considered acceptable (US EPA, 1989; USEPA, 2010). The results of this study showed that the carcinogenic risk for Cr, Ni, and Pb were acceptable or lower than the negligible level.

### 3.5 Benefit–risk ratio of the consumption of marine organisms based on the content of LC‐PUFA and toxic/essential elements

Values of benefit–risk hazard quotients (HQ_EFA_) based on the content of LC‐PUFA and toxic/essential elements are given in Table 6. A value of HQ_EFA_ below 1 indicates there is no risk for people to consume *M. galloprovincialis* (Gladyshev et al., 2009). The computation of the benefit (LC‐PUFA content) to risk (toxic/essential elements) ratio could be a more relevant way to estimate nutritive value of seafood rather than using concentration values alone. The content of trace elements in raw and steamed *M. galloprovincialis* did not decrease the nutritional value considering the benefit–risk ratio. There were no changes in the HQ_EFA_ for Cd, Cr, Cu, and Ni after the cooking process. However, steaming seems to decrease HQ_EFA_ for Fe (by 17%) and Pb (half‐fold), but increase for Zn. Nevertheless, raw and steamed *M. galloprovincialis* were characterized by very low HQ_EFA_ values («1), thus posing no risk for people consuming the daily portion providing the recommended intake of 500 mg/day EPA+DHA.

**TABLE 6:** Hazard quotients, HQ_EFA_, for benefit–risk ratio of LC‐PUFA (EPA + DHA) versus toxic/essential elements for intake of raw and steamed *Mytilus galloprovincialis* from the Black Sea

	Cd	Cr	Cu	Fe	Ni	Pb	Zn
Raw	0.47	0.04	0.02	0.06	0.002	0.02	0.06
Steamed	0.40	0.04	0.02	0.05	0.002	0.01	0.08

## 4 CONCLUSION

Thermal treatment method used for *M. galloprovincialis* culinary preparation affected all elements contents and fatty acid composition. Compared to the raw samples, steaming resulted in an increased concentration of Na, Mg, Zn, and saturated fatty acids and a decrease of polyunsaturated fatty acids. No effect on the DHA content was found. However, the significant increase in the absolute content of EPA+DHA indicates that steaming does not compromise the nutritional quality of mussels and people need to consume only 36–72 g of steamed mussels to meet the daily requirements. Both raw and steamed mussels exhibited beneficial antiatherogenic, antithrombogenic, and hypocholesterolemic indices. As all THQ, HI, and TR values were below 1, which lead to the conclusion that consumption of steamed *M. galloprovincialis* from the Bulgarian Black Sea did not offer any harm risk for the consumer's health concerning the analyzed toxic elements. Based on the benefit–risk ratio index (HQEFA), it can be concluded that steaming process did not significantly affect the nutritional quality of *M. galloprovincialis*. The benefits of *n*‐3 LC‐PUFAs intake provided by the regular consumption of steamed mussels outweigh the risk posed by the content of toxic and essential elements.

## CONFLICT OF INTEREST

The authors have no conflicts of interest to declare that are relevant to the content of this article.

## Data Availability

The datasets generated during and/or analyzed in the current study are available from the corresponding author on reasonable request.
